# Endohyphal Bacterium Enhances Production of Indole-3-Acetic Acid by a Foliar Fungal Endophyte

**DOI:** 10.1371/journal.pone.0073132

**Published:** 2013-09-24

**Authors:** Michele T. Hoffman, Malkanthi K. Gunatilaka, Kithsiri Wijeratne, Leslie Gunatilaka, A. Elizabeth Arnold

**Affiliations:** 1 School of Plant Sciences, The University of Arizona, Tucson, Arizona, United States of America; 2 Southwest Center for Natural Products Research and Commercialization, School of Natural Resources and the Environment, Tucson, Arizona, United States of America; University of Ottawa, Canada

## Abstract

Numerous plant pathogens, rhizosphere symbionts, and endophytic bacteria and yeasts produce the important phytohormone indole-3-acetic acid (IAA), often with profound effects on host plants. However, to date IAA production has not been documented among foliar endophytes -- the diverse guild of primarily filamentous Ascomycota that live within healthy, above-ground tissues of all plant species studied thus far. Recently bacteria that live within hyphae of endophytes (endohyphal bacteria) have been detected, but their effects have not been studied previously. Here we show not only that IAA is produced *in vitro* by a foliar endophyte (here identified as *Pestalotiopsis* aff. *neglecta*, Xylariales), but that IAA production is enhanced significantly when the endophyte hosts an endohyphal bacterium (here identified as *Luteibacter* sp., Xanthomonadales). Both the endophyte and the endophyte/bacterium complex appear to rely on an L-tryptophan dependent pathway for IAA synthesis. The bacterium can be isolated from the fungus when the symbiotic complex is cultivated at 36°C. In pure culture the bacterium does not produce IAA. Culture filtrate from the endophyte-bacterium complex significantly enhances growth of tomato *in vitro* relative to controls and to filtrate from the endophyte alone. Together these results speak to a facultative symbiosis between an endophyte and endohyphal bacterium that strongly influences IAA production, providing a new framework in which to explore endophyte-plant interactions.

## Introduction

Diverse plant-associated microbes synthesize phytohormones such as gibberellins, cytokinins, jasmonic acid, abscisic acid, ethylene, and indole-3-acetic acid (IAA), often with profound effects on growth, tissue differentiation, and reproduction of their hosts [1-8]. Microbial production of IAA is especially phylogenetically widespread, encompassing both plant-affiliated bacteria (e.g., *Erwinia* *herbicola* [9] and *Pantoea* *agglomerans* [10]) and diverse fungi ( *Mucoromycotina* , Basidiomycota, and Ascomycota [11-13]). Most examples of IAA production by plant-associated microbes come from plant pathogens, mycorrhizal fungi, rhizosphere endophytes, and bacteria and yeasts that are endophytic in above-ground tissues [14-19]. IAA produced by rhizosphere fungi can stimulate production of plant biomass, enhance growth rate of roots, and promote disease resistance [7,19,20]. In some cases, IAA produced by fungi affiliated with plants also can inhibit hypersensitive responses, reducing the production of defensive enzymes such as chitinase and glucanase (reviewed in 6).

In both natural and human-made environments, plants consistently harbor filamentous fungi (primarily Pezizomycotina, Ascomycota) in their apparently healthy above-ground tissues [21]. These endophytes (Class 3, sensu [21]; hereafter, endophytes) are known from every major lineage of land plants in biomes ranging from tundra to tropical forests [22-26]. They are transmitted horizontally and form numerous, localized infections in asymptomatic tissues such as leaves and stems [27-30]. At the community level they are highly diverse and individual plants frequently harbor multiple species, with significant turnover in endophyte assemblages over plant species’ ranges [23,25,26,31-33]. Endophytes often are closely related to pathogens, with transitions between endophytism and pathogenicity occurring frequently in the evolution of the Ascomycota [25].

Recent studies have shown that foliar endophytes frequently harbor highly diverse endohyphal bacteria of unknown importance (e.g., [34]). These bacteria occur in living hyphae of phylogenetically diverse endophytes isolated from various plant lineages and in multiple biogeographic provinces [34]. Those found in foliar endophytes are phylogenetically distinct from the apparently obligate symbionts of other plant-affiliated fungi (e.g., Glomeromycota) [34]. Phylogenetic analyses of endohyphal bacteria associated with filamentous foliar endophytes reveal no clear signal of fungal phylogeny, host plant phylogeny, or geography, suggesting a facultative association [34]. However, endohyphal bacteria of many fungi have not previously been cultivated independently of their hosts, and the effects of endohyphal bacteria on foliar endophytic fungi have not been evaluated to date. More broadly, the benefits or costs that endophytes and endophyte-bacterial complexes extend to their hosts, and the mechanisms by which endophytes with or without bacterial symbionts can escape, tolerate, or prevent induction of plant defenses remain major questions [24,28,34,35]. Because IAA is produced by diverse plant-associated fungi, can decrease host hypersensitive responses, and can enhance plant growth, we anticipated that IAA production could be an important but unexplored aspect of foliar endophyte-plant symbioses.

Here we provide the first documentation of IAA production by a foliar endophyte representing the Pezizomycotina (identified as *Pestalotiopsis* sp., with affinity for *Pe* *. neglecta* ) isolated from foliage of a coniferous host ( *Platycladus* *orientalis* , Cupressaceae). Further, we demonstrate that IAA production by the endophyte *in vitro* is enhanced significantly when the endophyte hosts an endohyphal bacterium (here identified as *Luteibacter* sp., Xanthamonadales). We show that IAA production by the endophyte and the endophyte-bacterial complex requires L-tryptophan. The bacterium, which can be cultured axenically, does not produce IAA on a standard growth medium. Culture filtrate from the endophyte-bacterium complex significantly enhances growth of a model plant (tomato) relative to controls and to filtrate from the endophyte alone, suggesting a potentially important but previously overlooked aspect of plant-endophyte symbioses.

## Materials and Methods

As part of a previous study [33], endophytic fungus 9143 was isolated on 2% malt extract agar (MEA) from surface-sterilized, asymptomatic foliage of a mature, healthy individual of *Platycladus* *orientalis* (Cupressaceae) in Durham, NC, USA. The isolate was archived as a living voucher in sterile water at the Robert L. Gilbertson Mycological Herbarium (ARIZ) at the University of Arizona (accession BA-9143). Previous phylogenetic analyses [34] confirmed placement of this isolate in the Xylariales but were insufficient to identify 9143 more definitively.

After observing its bacterial endosymbiont and IAA production (below), we identified the isolate as *Pestalotiopsis* aff. *neglecta* on the basis of conidial morphology after cultivation on MEA for 7 d (Figure S1) [36]. For confirmation, we compared sequence data from the nuclear ribosomal internal transcribed spacers and 5.8S gene of 9143, obtained by bidirectional Sanger sequencing in our previous work ( [33]; GenBank accession EF419899.1), with 35 sequences representing close relatives of *Pe* *. neglecta* [37-41], which were obtained from GenBank. The dataset, including *Seiridium* as the outgroup, was aligned automatically with default parameters in ClustalW 1.0 [42] and adjusted manually in Mesquite v. 1.06 [43]. The best-fitting model of evolution (GTR+I+G) was inferred using Modeltest 3.7 [44]. Bayesian analysis was implemented in MrBayes v. 3.1.2 [45] with 2 sets of 5 million generations each, initiated with random trees, four chains, and sampling every 1000^th^ tree. After elimination of the burn-in, defined by assessment of -ln li values, the remaining trees were used to infer a majority rule consensus. A complementary maximum likelihood (ML) inference was conducted with GARLI v1.0 [46] using default settings and GTR+I+G, followed by bootstrap analysis (100 replicates).

### Identification of endohyphal bacterium

Previous analyses revealed that endophyte 9143 harbored an endohyphal bacterium, which we identified previously on the basis of 16s rRNA sequencing and light microscopy as a member of the Gammaproteobacteria ( [34]; GenBank accession HM117737). The 16S rRNA sequence obtained by bidirectional Sanger sequencing using primers 27F and 1492R [47] was aligned manually in Mesquite v. 1.06 [43] with sequences of closely related Xanthomonadales obtained from GenBank. Bayesian analysis was implemented in MrBayes v. 3.1.2 [45] on the CIPRES teragrid portal [48] for 2 runs of 10 million generations each, initiated with random trees, four chains, and sampling every 1000^th^ tree, using GTR+I+G based on evaluation in Modeltest 3.7 [44]. After elimination of the burn-in as described above, the remaining trees were used to infer a majority rule consensus. Complementary maximum parsimony (MP) and neighbor joining (NJ) analyses were performed in PAUP* 4.0 [49]. The MP heuristic search included random stepwise addition, tree bisection-reconnection (TBR), and gaps treated as missing data. Branch support was assessed using a nonparametric NJ bootstrap [50] and Bayesian posterior probabilities. Phylogenetic placement was consistent with *Luteibacter* sp., Xanthomonadaceae, Xanthomonadales, Gammaproteobacteria (see below).

### Production of bacterium-free clone

Three replicate subcultures of endophyte 9143 were cultivated on 2% MEA, and on 2% MEA amended with 40 µg ml^-1^ of the antibiotic ciprofloxacin, for 10 d. Bacterial infection status in fresh mycelium was evaluated using light microscopy (400X), which ruled out external contaminants; Live-Dead stain, which established that bacteria and hyphae were viable; and DNA extraction, 16S rRNA PCR, and sequencing per above, which confirmed the presence or absence of *Luteibacter* following [34]. Cultures with (hereafter, 9143+) and without (9143-) the endohyphal bacterium were stored separately at -80 °C in 80% glycerol. Growth rates of 9143+ and 9143- were compared in triplicate on 2% MEA and water agar at 22°C and 36°C, and at three pH levels on MEA (pH = 4.5, 6.8, and 8.0) as described in [50].

### Measurement of indole compound production *in vitro*

Small (2mm^2^) fragments of mycelium from isolates of 9143+ and 9143- were plated on 2% MEA and allowed to grow for 7 d. Pieces removed from resulting cultures with a sterile cork borer were used to inoculate three flasks containing 80ml of Czapek Dox broth (CDB; Hymedia; pH 7.2) augmented with L-tryptophan (Sigma; 5 mM). For each treatment set, one flask containing only sterile CDB was used as a negative control. Flasks were checked for contamination after agitating at 120 rpm at 26 °C for 24 h.

After 72 h, three 1 ml aliquots were removed from each flask and centrifuged to remove cells from suspension. The Salkowski colorimetric technique then was used to estimate the concentration of indole compounds by treating the supernatant with 2 ml of Salkowski reagent (1 ml, 0.5 mM FeCl_3_ and 50 ml, 35% HClO_4_) [51-53]. Samples were incubated at room temperature for 30 min, and then evaluated at 530 nm on a spectrophotometer. CDB supplemented with L-tryptophan (as above) was used as a blank. IAA concentrations were determined with an IAA standard curve using commercial IAA (Sigma) and sterile medium as a blank.

### Identification of the indole compound as IAA

We used thin layer chromatography (TLC) and high performance liquid chromatography (HPLC) to identify the indole compound revealed by the diagnostic colorimetric reaction with Salkowski reagent. Two cultures each of 9143+ and 9143- were grown for 14 d in 500 ml of sterile CDB supplemented with 5 mM L-tryptophan. One additional culture of each was grown in sterile CDB without L-tryptophan (1 L) to evaluate whether the pathway for IAA production is tryptophan-dependent. Each culture was filtered using Whatman No. 1 filter paper. Each filtrate (1 L) was extracted three times with 500 ml of ethyl acetate (EtOAc). The combined EtOAc layer was washed three times with 500 ml of H_2_O, dried over anhydrous Na _2_SO_4_, and evaporated under reduced pressure to yield an EtOAc extract.

Normal phase TLC analysis of the EtOAc extract was performed on aluminum-backed plates coated with a 0.20 mm layer of silica gel 60 F_254_ (E. Merck, Darmstadt). Spots were visualized by inspection of plates under UV (254 nm) and after spraying with Van Urk-Salkowski reagent [52] followed by heating. Analytical reversed phase TLC investigations were performed on aluminum-backed plates coated with a 0.20 mm layer of silica gel 60 RP-18 F_254_S (E. Merck, Darmstadt; eluant: 20% MeCN in H_2_O, *R*_*f*_ 0.5). Analytical HPLC analysis of the EtOAc extracts was performed using a Kromasil 5 μm C-18 column (4.6 x 250 mm) on a Shimadzu HPLC system equipped with DGU-14A degasser, LC-10AD*vp* pump, SPD-M10A*vp* diode array detector and SCL-10A*vp* system controller utilizing Shimadzu LC-MS solution software. Samples were redissolved in MeOH (2.0 mg ml^-1^) and injections (10 μl) were made with Shimadzu SIL-10AD*vp* auto injector. The mobile phase consisted of H_2_O/MeCN/HCOOH (69.75:30.00:0.25) with a flow rate of 1.2 ml min^-1^. Commercial IAA (Sigma) was used as an authentic sample (eluant: 8% MeOH in CH_2_Cl_2_, *R*_*f*_ 0.3), with peak enhancement following injection confirming identity of putative IAA peaks in each analysis.

### Seedling assays

Isolates of 9143+ and 9143- were grown for 14 d in 200 ml CDB with 5 mM L-tryptophan as described above and then vacuum-filtered through a 0.44 µm nylon filter. The pH of each filtrate (pH = 5.7 for 9143+; pH = 4.2 for 9143-; pH = 7.1 for CDB alone) was amended to 7.0-7.1 with 0.5 M NaOH as needed. Twenty-five ml of each filtrate was further filter-sterilized using 0.2 µm syringe filters for seedling assays.

Tomato seeds (ACE 55; The Home Depot) were surface-sterilized by agitating in 50% bleach for 12-15 minutes, rinsed in sterile water, and placed on sterile filter paper with 3ml of sterile water in 60 mm Petri dishes (20 seeds/dish). Plates were sealed with Parafilm and seeds were allowed to germinate at 25 °C for 5 d. Sets of 10 apparently healthy seedlings then were chosen randomly and transferred under sterile conditions to new 60 mm plates containing sterile filter paper. Each set of seedlings was treated with 3.5 ml of sterile water. Four plates per treatment (i.e., 40 seedlings/treatment) then received 50 µl per seedling of one of five treatments: (a) filter-sterilized CDB from 9143+; (b) filter-sterilized CDB from 9143-; (c) filter-sterilized CDB + 5 mM L-tryptophan; (d) filter-sterilized CDB + commercial IAA (0.1 mg ml^-1^; pH 7.0); or (e) sterile water. Plates were sealed with Parafilm and incubated under fluorescent lights at room temperature for 5 d (12 h light/dark cycle). At harvest seedlings were stretched to full length by mounting to paper with transparent cellophane tape. Shoot and root lengths to longest points were measured using calipers. Data were analyzed in JMP^®^ 8.0 using ANOVA after normalizing all measurements to the CDB + IAA treatment (treatment d).

### Isolation of the endohyphal bacterium

Incubating 9143+ on 2% MEA at 36 °C for 7 d resulted in emergence of bacterial growth from the fungal mycelium. We sequenced 1100 bp of the 16S rRNA region of the emergent bacterium (BAC182) as above (see also 47). The sequence was identical to that of the bacterium sequenced directly from genomic DNA of endophyte 9143+. BAC182 was grown overnight in sterile LB broth and vouchered in sterile glycerol at -80 °C. The bacterium was screened for IAA production as described above.

## Results

**Figure 1:**
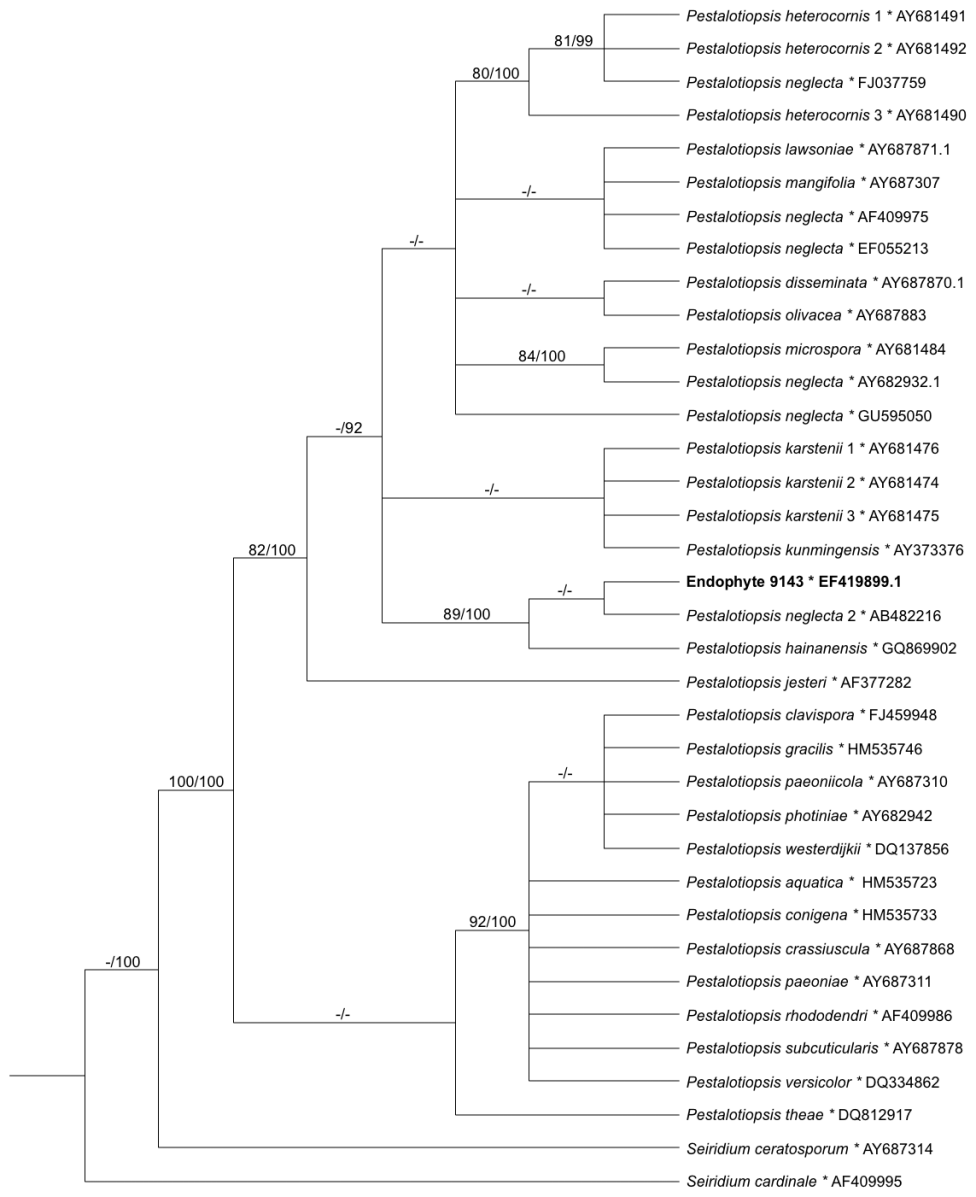
Majority rule consensus based on Bayesian analyses of ITSrDNA sequences representing *Pestalotiopsis* spp. with affiliation for *Pe. neglecta*, with *Seiridium* as the outgroup. Values indicate maximum-parsimony bootstrap ≥70% (before slash) and Bayesian posterior probability ≥90% (after slash).

**Figure 2:**
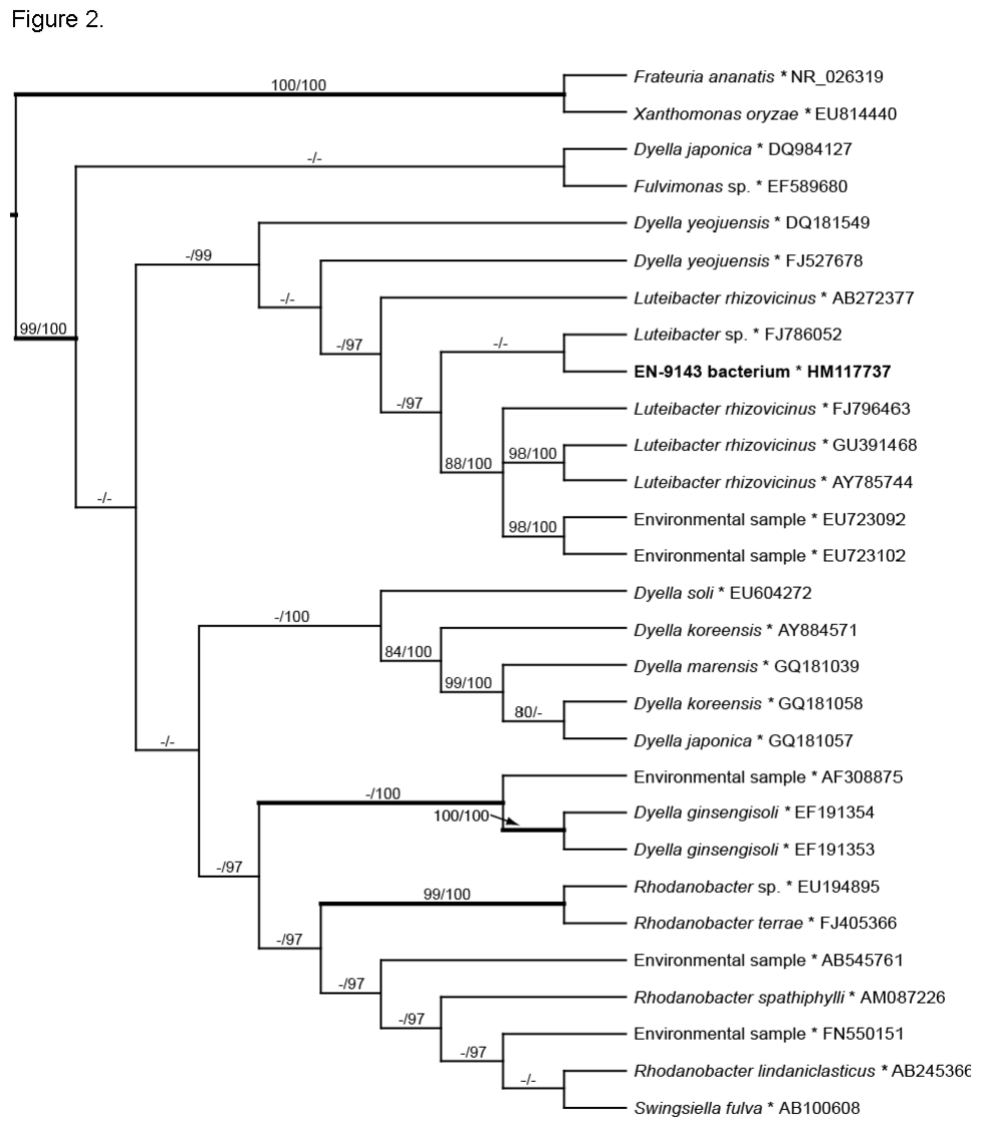
Majority rule consensus based on Bayesian analyses of 16S rRNA sequences of selected Xanthomonadales with affinity for the endohyphal bacterium from endophyte 9143. Values indicate maximum-parsimony bootstrap ≥70% (before slash) and Bayesian posterior probability ≥90% (after slash). Branches in bold indicate neighbor-joining bootstrap values ≥70%. *Swingsiella* *fulva* = *Rhodanobacter* *fulvus*.

After cultivation on 2% MEA, conidia of endophyte 9143 were observed as multisetulate, fusiform-shaped, and concolorous, without knobbed appendages (see 36). Phylogenetic analyses placed endophyte 9143 as sister to one of three taxa identified as *Pe* *. neglecta* by [40], congruent with morphological assessment (Figure 1, S1). Our results are congruent with previous studies (e.g., [40]) in placing two other putative “ *Pe* *. neglecta* ” sequences, which appear to represent misidentified sequences from GenBank, in a separate subclade. Based on morphology and phylogenetic placement relative to a recognized and vouchered isolate of *Pe* *. neglecta* , we consider 9143 to be *Pestalotiopsis* aff. *neglecta*, with further systematic revision to follow. In turn, phylogenetic analyses of 16S rRNA confirmed placement of the endohyphal bacterium from 9143 in the Xanthomonadaceae, with strong support within *Luteibacter* (*Luteibacter* sp.; Figure 2).

We found no difference between 9143+ and 9143- isolates in growth rate on 2% MEA or water agar at 22 °C, nor with regard to pH of the growth medium (Figure S2). Neither 9143+ nor 9143- grew at 36 °C, but cultivation of 9143+ at this temperature resulted in successful isolation of the endohyphal bacterium (above).

**Figure 3:**
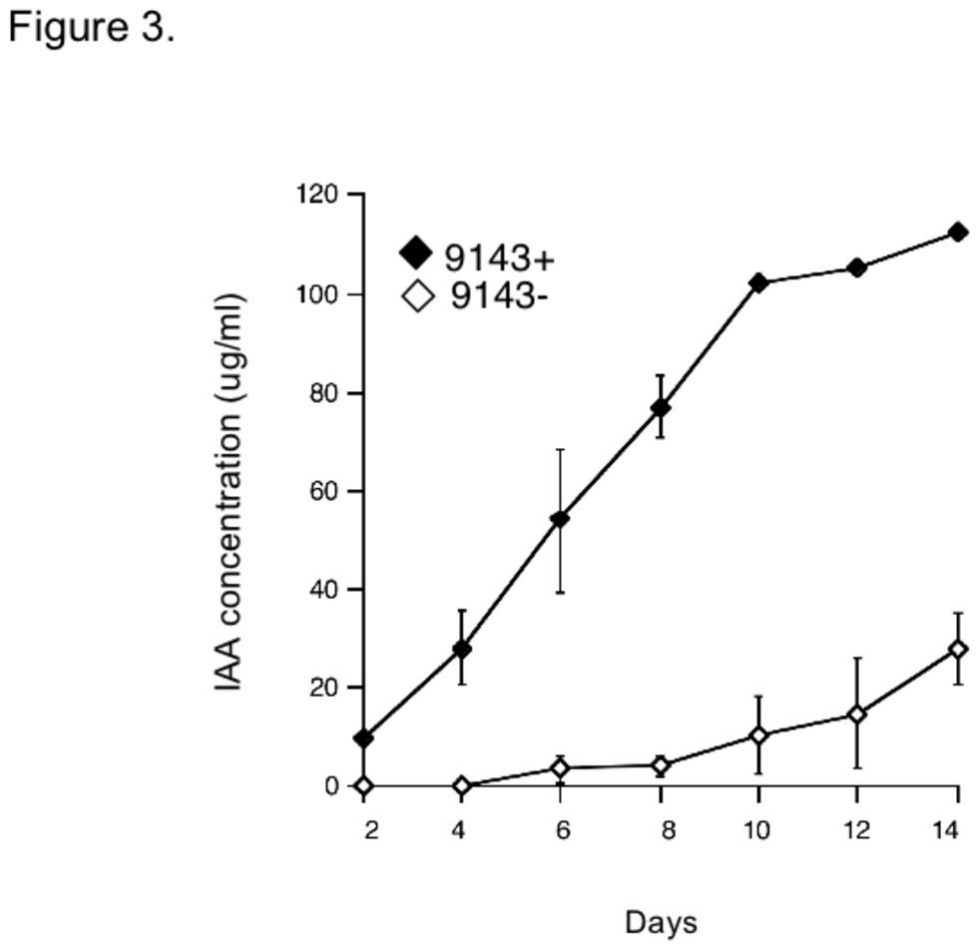
IAA concentrations estimated using the Salkowski colorimetric technique, measured as spectrophotometric absorbance at 530nm following treatment of supernatant from CDB cultures of 9143 containing the endohyphal bacterium (9143+) and lacking the bacterium (9143-). Points represent the mean and standard deviation of three readings (two-tailed *t*_4_ = 20.99; *p* < 0.0001), converted to concentration using the IAA standard curve (y = 0.0108x -0.0049).

Chromogenic testing indicated that 9143- produced an indole compound when growing axenically *in vitro* (Figure 3). Production of that indole compound was enhanced significantly when the mycelium of 9143 contained the endohyphal bacterium (9143+; repeated measures ANOVA, *F*_1, 4_ = 358.7; *p* < 0.0001; Figure 3). After 14 d, mean concentration of the indole compound in CDB from 9143+ (104.8µg ml^-1^) was 78 µg ml^-1^ greater than that produced by 9143- grown under the same conditions (Figure 3).

**Figure 4:**
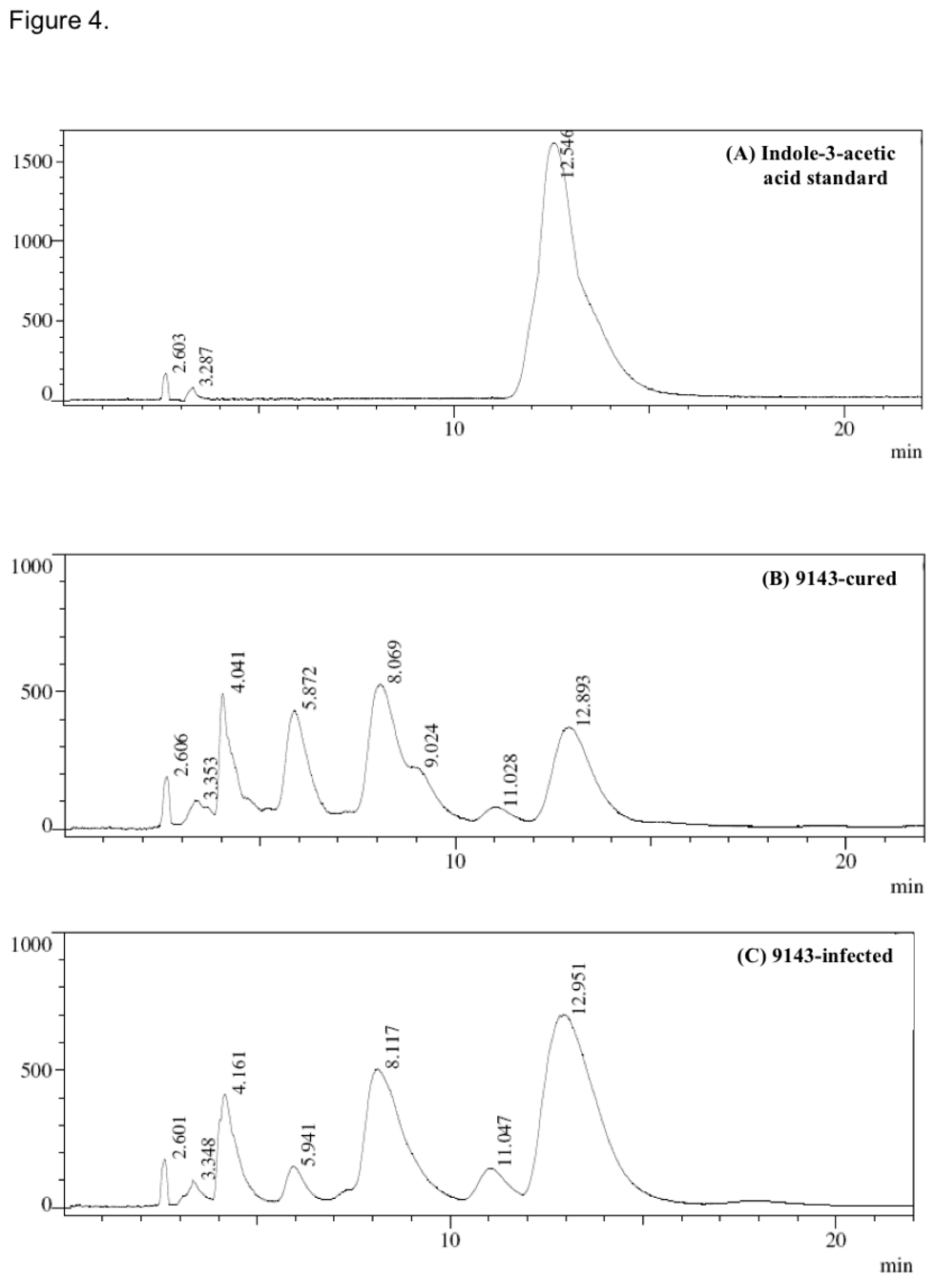
HPLC results (retention time and co-injection) for (A) IAA standard, and filtrate extracts from (B) 9143+ and (C) 9143-. Elution time was 12.54 minutes for the IAA standard, 12.89 for 9143+, and 12.95 for 9143-. Slight differences in retention times reflect partial masking due to constituents in the sample matrix, a known feature of HPLC analysis using reversed-phase columns.

TLC and HPLC identified the indole compound as indole-3-acetic acid (IAA), and confirmed that 9143 produced significantly more IAA when the endohyphal bacterium was present vs. absent (Figure 4). No IAA was produced when 9143+ and 9143- isolates were grown in CDB without L-tryptophan, nor by *Luteibacter* when grown alone in CDB + tryptophan (data not shown).

**Figure 5:**
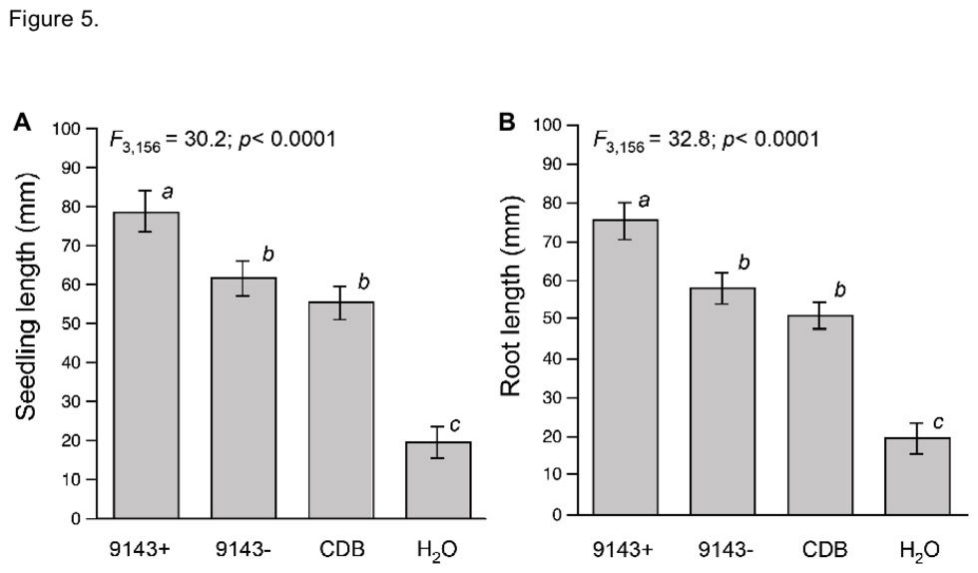
Growth responses of tomato seedlings to supernatant from 9143+ cultures in CDB; supernatant from 9143- in CDB; and control treatments (CDB alone, water). (A) Total seedling length, (B) root length. Data were normalized to measurements for plants that received CDB + IAA. Different superscripts within each panel indicate significantly different means.

Tomato seedlings treated with filter-sterilized CDB from 9143- did not differ significantly in root length or total seedling length relative to the CDB control (Figure 5, post-hoc Tukey-Kramer test, alpha = 0.05). However, seedling length was significantly increased by treatment with CDB from 9143+, reflecting significantly longer roots relative to controls and treatment with CDB from 9143- (Figure 5, Tukey-Kramer tests, alpha = 0.05).

## Discussion

Endohyphal bacteria have been found previously in living hyphae of plant-associated Glomeromycota, *Mucoromycotina* , and several ectomycorrhizal Dikarya (*Tuber borchii*; Ascomycota; *Laccaria* *bicolor* and *Piriformospora* *indica* ; Basidiomycota) [54-59,61,62]. Recently, we reported their presence in foliar endophytic fungi representing four classes of Pezizomycotina, and demonstrated that they are both geographically widespread and phylogenetically diverse [34]. The present study is the first to identify the partners in a close ecological relationship between a foliar endophyte and an endohyphal bacterium, and the first to show that (a) a species of *Pestalotiopsis* (here identified as *Pestalotiopsis* sp. aff. *neglecta*) is capable of producing a phytohormone; (b) a filamentous endophyte from above-ground tissues can produce indole-3-acetic-acid (IAA); (c) IAA produced by this endophyte appears to be dependent on the L-tryptophan pathway; (d) an endohyphal bacterium (*Luteibacter* sp.) significantly enhances this IAA production, but does not produce measurable IAA when grown axenically; and (e) exogenous culture filtrate of the endophyte with its endohyphal symbiont significantly enhances root growth of a model plant *in vitro* relative to controls and to filtrate from the endophyte alone. Moreover, our work shows that the endohyphal bacterium affiliated with 9143 can be isolated following heat-treatment of the host endophyte in culture.

The lack of IAA production by *Luteibacter* sp. in axenic culture contrasts with the ample production (ca. 40 µg ml^-1^ of IAA) by axenic *Rhizobium radiobacter*, a culturable bacterial endosymbiont associated with the root endophyte *Piriformospora* *indica* (Sebacinales, Basidiomycota) [59]. It is possible that production could be observed by altering the growth medium on which *Luteibacter* was grown for the present work, perhaps through enhancement of tryptophan availability.

In the presence of endohyphal *Luteibacter* sp. and L-tryptophan, 9143+ cultivated in CDB produced ca. 100 μg ml^-1^ of IAA. This amount is likely biologically significant, as suggested by seedling assays and by previous studies with plant-pathogenic and rhizosphere fungi: IAA production observed here was within the range produced by *Ustilagomaydis* (75-262 μg ml^-1^) as measured using the same colorimetric reagents [15]. Using GC-MS and HPLC-ESI_MS/MS, culture filtrate from *Piriformospora* *indica* yielded more IAA than was observed here [7]. In turn, isolates of *Colletotrichum* were found to generate 2-32 μg ml^-1^ of IAA using TLC and GC-MS [5,17], consistent with the values observed in 9143-. 

Recent work has shown that closely related or conspecific fungi can harbor different endohyphal bacteria [34]. These associations appear to be lost readily, as observed under heat treatment (above). The mechanism by which they are acquired has not yet been determined for foliar endophytes, although spore invasion in soilborne Glomeromycota has been observed [57]. Our results, coupled with phylogenetic analyses of endohyphal bacteria that reveal no signal of fungal phylogeny, host plant phylogeny, or geography [34], are consistent with a facultative association that contrasts with the obligate association between endohyphal bacteria and some arbuscular mycorrhizal fungi [54].

Although our study does not yet address bacterial-fungal-plant interactions during the endophyte symbiosis, it provides a first estimation of one way in which an apparently facultative bacterial endosymbiont can influence interactions between an endophytic fungus and its host. We attribute the observed differences in root growth to enhancement of IAA production by the endophyte/endohyphal bacterium relative to the endophyte alone, in part because of the large magnitude of change in IAA (Figures 3, 4) relative to other compounds (Figure 4). It is possible that other compounds detected by HPLC (Figure 4) might selectively inhibit growth; this is a focus for future study. In the meantime we are interested to assess whether IAA production by the endophyte or endophyte/endohyphal bacteria complex can decrease host defensive responses to this and related endophytes [34], thus contributing substantively to the capacity of endophytic fungi to grow asymptomatically host tissue.

### Conclusions and perspectives

Our study provides the first evidence that bacterial associates of foliar endophytes can influence phytohormone production. We predict that the facultative nature of the endohyphal symbiosis may account for some of the diversity and ecological plasticity observed in endophyte-plant interactions [25]. More generally, increasing but still limited exploration of ectohyphal bacteria, endohyphal bacteria, and mycoviruses has begun to illustrate the powerful but often overlooked ways in which microbes associated with fungal hyphae can influence the outcome of plant-fungus associations [19,60-62]. Advancing our knowledge of endophyte interactions with plant hosts, other extrinsic microorganisms, and most recently, diverse endohyphal bacteria, will help define the functional biology of these diverse and ubiquitous symbionts.

## Supporting Information

Figure S1 **

*Pestalotiopsis* *neglecta* asexual spore morphology coincides with the morphology of conidia from endophyte 9143.** Conidia are fusiform, four-septate, a fuliginous brown in color, with end cells hyaline. The apical end is short with two or three spreading setulae, approximately 22 um long. The basal end contains a pedicel about 4-7 um long (Steyaert, 1953). Image depicts conidia from 9143- (400X) following cultivation on 2% MEA, showing fusiform cells with 4 septae.
(TIFF)

Figure S2 **Results of growth assays over 14 days for 9143+ (black diamond) and 9143- (open square) on 2% MEA at pH = 4.5 (panel A), pH = 8.0 (panel B), and pH = 6.8 (standard 2% MEA; panel C) at 22°C.** Panel D shows growth on water agar at 22 °C. 9143 did not grow at 36 °C, such that data are not shown. Error bars indicate standard error of the three replicates performed for all experiments.
(TIFF)
