# Supplementary figures and images for: Endohyphal Bacterium Enhances Production of Indole-3-Acetic Acid by a Foliar Fungal Endophyte
Source: PLoS One. 2013 Sep 24;8(9):e73132. doi: 10.1371/journal.pone.0073132 (PMC3782478; doi:10.1371/journal.pone.0073132)

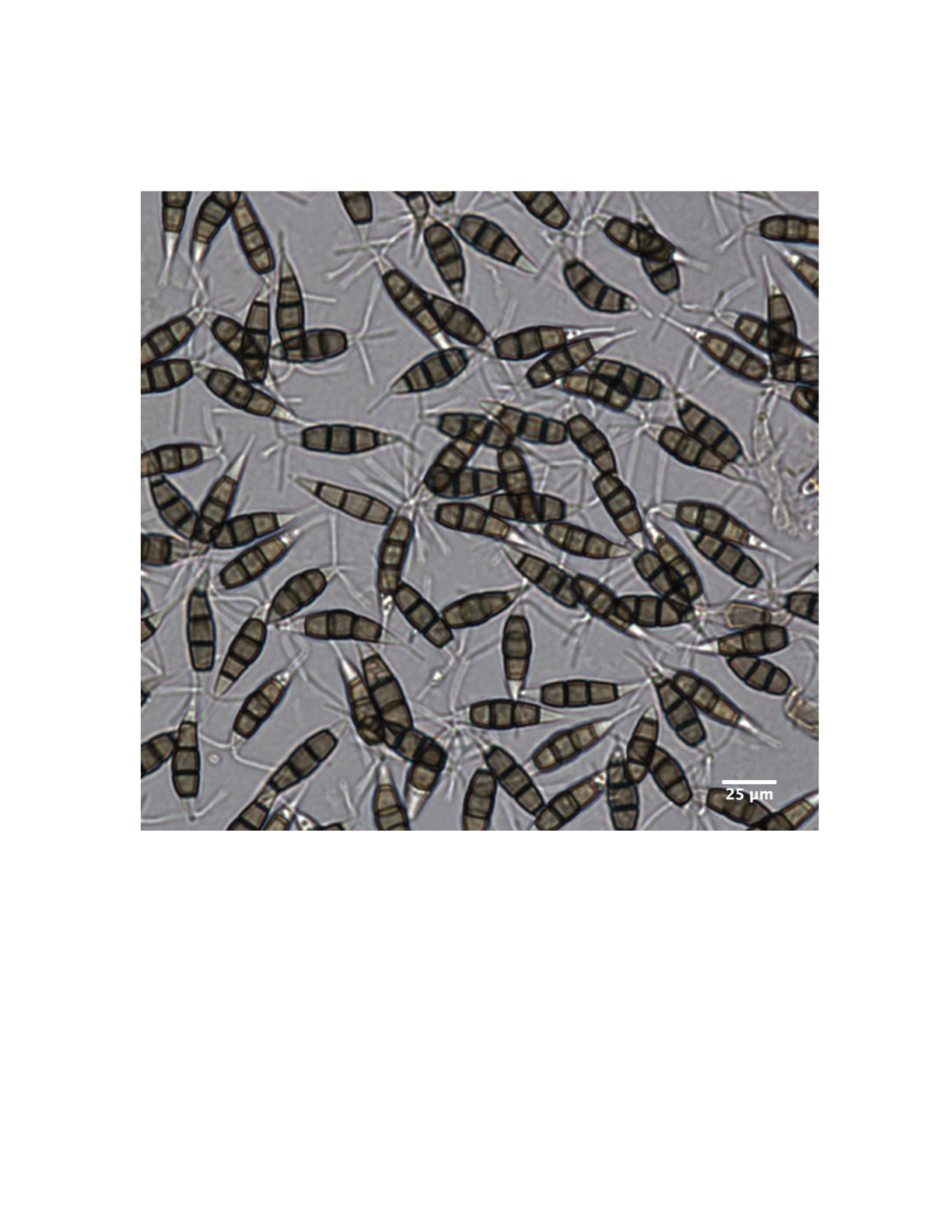

Supplement: Figure S1 — Pestalotiopsis neglecta asexual spore morphology coincides with the morphology of conidia from endophyte 9143. Conidia are fusiform, four-septate, a fuliginous brown in color, with end cells hyaline. The apical end is short with two or three spreading setulae, approximately 22 um long. The basal end contains a pedicel about 4-7 um long (Steyaert, 1953). Image depicts conidia from 9143- (400X) following cultivation on 2% MEA, showing fusiform cells with 4 septae. (TIFF) [file pone.0073132.s001.tiff]

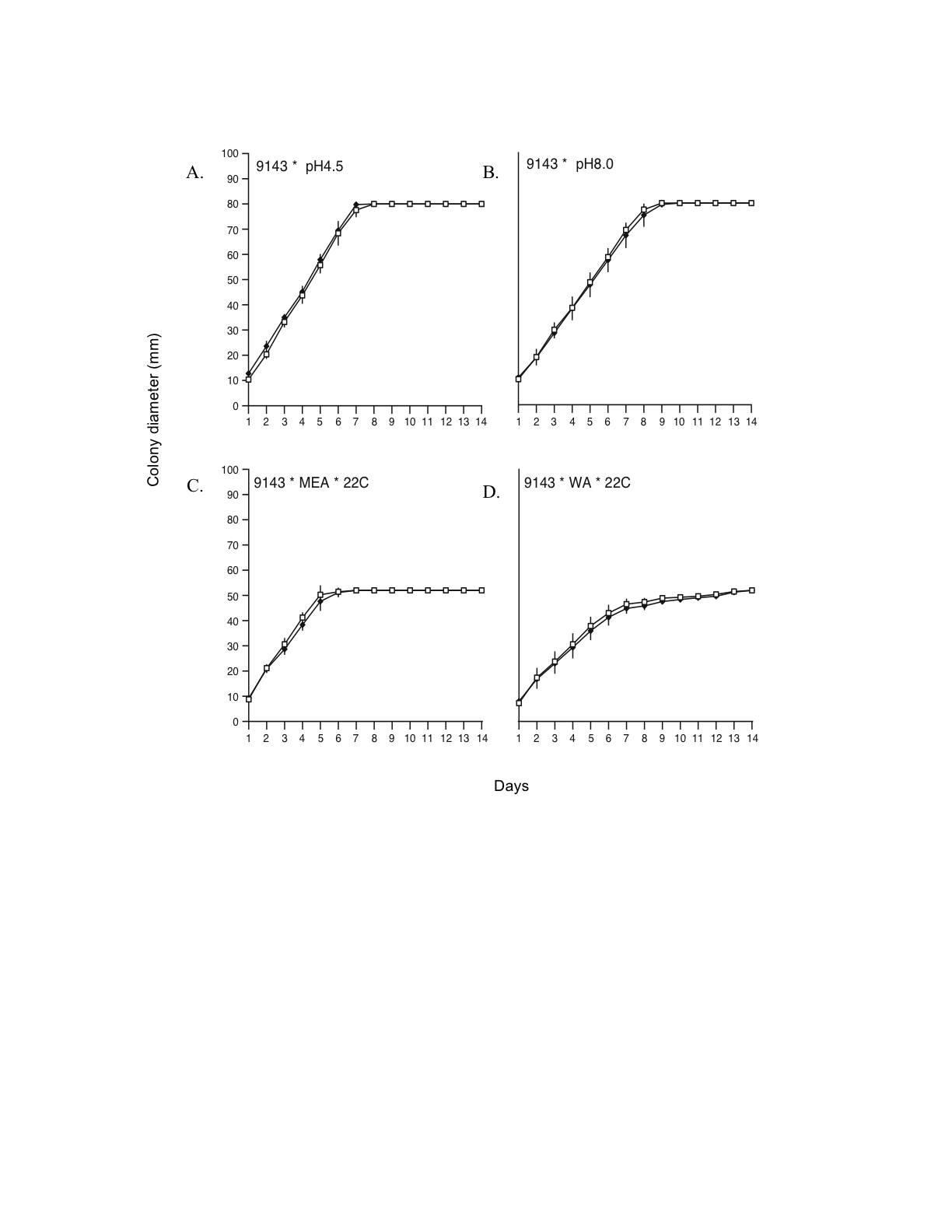

Supplement: Figure S2 — Results of growth assays over 14 days for 9143+ (black diamond) and 9143- (open square) on 2% MEA at pH = 4.5 (panel A), pH = 8.0 (panel B), and pH = 6.8 (standard 2% MEA; panel C) at 22°C. Panel D shows growth on water agar at 22 °C. 9143 did not grow at 36 °C, such that data are not shown. Error bars indicate standard error of the three replicates performed for all experiments. (TIFF) [file pone.0073132.s002.tiff]
